# Prospective associations between measures of gross and fine motor coordination in infants and objectively measured physical activity and sedentary behavior in childhood: Erratum

**DOI:** 10.1097/MD.0000000000010013

**Published:** 2018-02-16

In the article, “Prospective associations between measures of gross and fine motor coordination in infants and objectively measured physical activity and sedentary behavior in childhood”,^[1]^ which appeared in Volume 96, Issue 46 of *Medicine*, Dr. Justin Roberts name appeared incorrectly.
